# Autonomous path planning for intercostal robotic ultrasound imaging using reinforcement learning

**DOI:** 10.1038/s41598-026-37702-9

**Published:** 2026-02-11

**Authors:** Yuan Bi, Cheng Qian, Zhicheng Zhang, Nassir Navab, Zhongliang Jiang

**Affiliations:** 1 Chair for Computer Aided Medical Procedures (CAMP), Technical University of Munich, Munich, Germany; 2 School of Computer Science,Carnegie Mellon University, Pittsburgh, USA

**Keywords:** Computational biology and bioinformatics, Engineering, Mathematics and computing, Medical research

## Abstract

Ultrasound (US) is widely used in clinical practice for the screening internal organs and guiding interventions. Nonetheless, US imaging suffers from inter- and intra-operator variations. Leveraging the reproducibility offered by robots, robotic ultrasound systems emerge as a promising solution, offering enhanced precision, stability, and repeatability. To realize autonomous US scanning, robot learning algorithms have been widely explored. However, current approaches primarily base the decision-making process for US navigation on 2D US images data, often overlooking the integration of 3D anatomical knowledge, which is a critical component for path planning in anatomically complex regions, such as the intercostal area. To address this limitation, we propose a novel reinforcement learning (RL) approach for intercostal US scanning path planning, leveraging computed tomography (CT) templates and utilizing 3D state representations. To this end, a virtual environment is developed using CT templates with randomly initialized tumors of various shapes and locations as a training environment. In addition, task-specific state representation and reward functions are introduced to encourage the convergence of the training process while minimizing the effects of acoustic attenuation and shadows during scanning. It is important to note that the scope of this work is limited to the autonomous path planning component, while robotic execution and control integration will be addressed in future studies. To validate the effectiveness of the proposed approach, experiments have been carried out on unseen patient models with randomly defined single or multiple scanning targets. The results demonstrate the efficiency of the proposed RL framework in planning non-shadowed US scanning trajectories in areas with limited acoustic access.

## Introduction

**Fig. 1:**
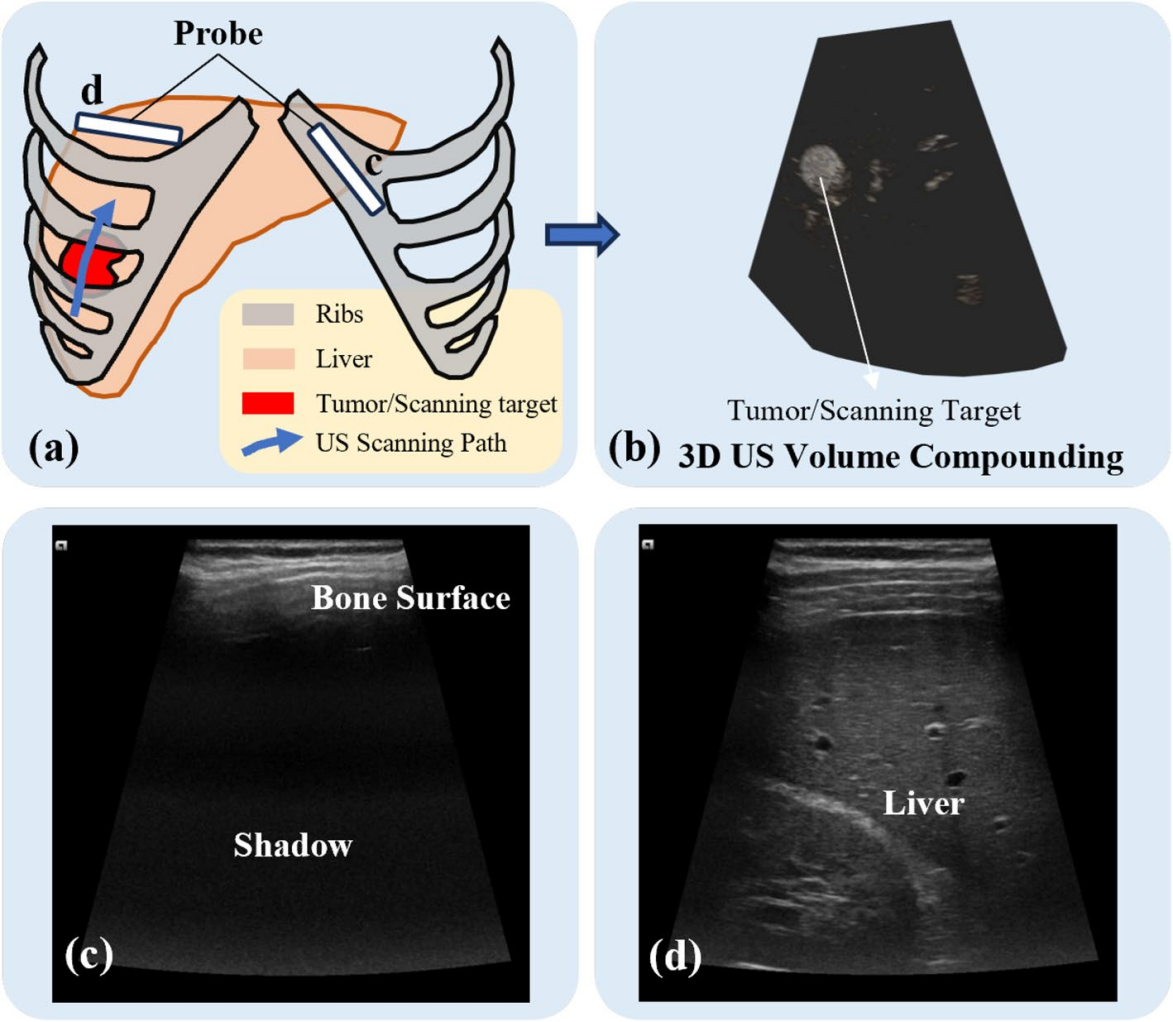
(**a**) Our objective is to plan a US scanning trajectory to fully cover a specific area beneath the ribs during biopsy or ablation procedures. Such area can be an already identified tumor or some suspicious regions defined by the doctors based on CT images. Although CT provides preoperative information, real-time US imaging is essential for intraoperative guidance, allowing continuous monitoring of the target area during the procedure. (**b**) Combining with the tracking information, the final goal is to realize the US reconstruction of the selected area. The planned trajectory should avoid the occlusion of bones and try to perform the US acquisition through intercostal gaps. Two representative probe positions are illustrated in (**a**), with the corresponding US images displayed in (**c**) and (**d**).

Ultrasound (US) imaging has been one of the most widely used modalities in clinical practice, e.g., for fetuses^1^, internal organs^2^, and vessels^3^. Compared to computed tomography (CT) and magnetic resonance imaging (MRI), US offers advantages, including low cost, high portability, and real-time imaging capabilities. Leveraging its real-time nature, US is frequently used for needle guidance in biopsy and ablation procedures (e.g., liver, prostate, and breast interventions). As a portable device, US gives operators high flexibility during examinations, but in the meantime, it also hinders the standardization of image acquisitions. A lot of factors can affect the quality of US images, including internal (frequency of US wave, dynamic range, focus, etc.) and external acquisition parameters (applied force and pose of the probe)^4–6^. In addition, to monitor the anatomy of interest covered by the rib cage (e.g., liver or heart), a proper scanning path in the limited intercostal space is needed to avoid acoustic shadows cast by bone structures [see Fig. 1 (c)]. For instance, during and after the liver ablations, the surgeons need to monitor the tumor as a whole to correctly assess the surgical outcomes. This task requires specific training to understand both the US images and underlying anatomical knowledge. Therefore, inter- and intra-operator variations often arise.

Robotic systems have demonstrated superior performance in terms of precision, stability, and reproducibility. Research on robotic ultrasound systems (RUSS) presents a promising solution to mitigate operator-dependent variations^7,8^. According to ultrasound propagation principles, maintaining the probe orthogonal to the surface maximizes the capture of reflected acoustic signals, enhancing visualization quality (e.g., contrast)^9,10^. However, this principle does not apply straightforwardly to thoracic applications, where the visibility of internal organs is significantly obstructed by acoustic shadows caused by bones with high acoustic impedance. Several rule-based path planning methods have been proposed using external cameras^11–13^ or preoperative CT scans^14^. While these methods demonstrate robustness in specific applications, automatically generating an optimal US scanning trajectory for comprehensive volume coverage under the ribs–while avoiding acoustic shadowing–remains a significant challenge due to the complexity of the intercostal region.

To address this challenge, reinforcement learning (RL) has emerged as a promising alternative solution for serial decision-making problems. RL operates by interacting with the environment to maximize task-specific reward functions. In the context of RUSS, RL has been employed to identify standard US views containing diagnostically meaningful information^15–18^. However, existing methods primarily focus on detecting standard imaging planes rather than planning a scanning trajectory to reconstruct and monitor lesion changes (e.g., liver tumors) beneath the rib cage. In addition, these studies rely on partial observations, such as US images, contact force, and action history, rather than the true state, which includes the relative position between the probe and the scanned anatomical structures. Integrating anatomical knowledge as prior information and mapping real-time observations onto a virtual model, similar to clinicians’ mental representations, can transform a partially observable problem into a fully observable one. This approach significantly reduces task complexity, making it especially useful for complex navigation tasks of liver with limited intercostal acoustic windows.

To generate an optimal intercostal scanning path for visualizing target volumes beneath the ribs, this study introduces an RL framework trained using CT-based templates. To effectively represent the state at each step, the framework incorporates 3D scene representations of the rib cage, the scanning target (i.e., liver tumors), and the US imaging plane. By leveraging 3D scene views, the problem is formulated as a fully observable Markov decision process (FOMDP), providing global contextual information. This transformation enhances the robustness of the trained policy in identifying an optimal scanning trajectory. Compared with existing rule-based or image-based robotic ultrasound methods, the proposed framework uniquely integrates CT-derived anatomical knowledge into the reinforcement learning formulation. This enables the agent to reason about spatial relationships among the ribs, target structures, and ultrasound beam geometry rather than relying solely on 2D image cues. In addition, the use of task-specific state and reward designs allows the model to jointly optimize target coverage, attenuation minimization, and shadow avoidance–three essential criteria for effective intercostal ultrasound path planning. The main contributions of this study are as follows:

- An RL-based method is proposed to tackle the challenging task of intercostal US scanning path planning. Instead of US image guided trajectory planning, this approach uses CT atlases to cast a partially observable decision-making problem into a fully observable one. Task-specific reward functions and state representations are proposed to ensure full coverage and reconstruction of single or multiple regions of interest while minimizing the acoustic attenuation and shadows.
- A simulation environment for US screening of internal organs or lesions through intercostal spaces has been developed based on the CT atlas. A cylindrical coordinate system is employed to efficiently model the 6D movements of US probes in the vicinity of the ribcage region and ensure the adaption of patients with varying body sizes.

The effectiveness of the proposed framework has been validated using publicly available, previously unseen patient data^19^. As a next step, to enable the transfer of the planned path from CT templates to individual patients for autonomous robotic scanning, a non-rigid skeleton graph-based registration algorithm^20,21^ can be integrated. However, it is important to note that this study primarily focuses on the robust and autonomous generation of scanning trajectories. The code will be released upon acceptance.

## Related work

### Path planning for RUSS

In order to guarantee the full coverage of the anatomy of interest, Wang *et al.* introduced a path planning pipeline for breast US scanning based on point clouds captured by an external RGB-D camera^22^. Yang *et al.* utilized a deep learning network to extract the spine area of patients from camera input to pre-plan the US sweep trajectory^23^. Besides external cameras, CT and MRI are also frequently utilized for scanning path planning^24^. Hennersperger *et al.* proposed a registration-based method to transfer the scanning path planed on pre-operative MRI/CT images to patients^25^. Jiang *et al.* implemented an MRI atlas-based non-rigid registration framework to address the articulated motions of human arms^26^. These methods work robustly in their cases, while their effectiveness is decayed for thoracic applications due to the occlusion of rib cage.

In order to image the whole liver, Mustafa *et al.* localized the region of interest by extracting umbilicus and mammary papillae from a web camera and then the probe was controlled to perform wobbler motion to obtain a wider view^2^. However, its performance in intercostal spaces is limited without explicitly considering the bone occlusion. To address this challenge, Göbl *et al.* developed an automatic acoustic-window planning method based on patient-specific CT data to determine feasible, shadow-free probe poses for a given target point using rule-based geometric constraints^14^. Al-Zogbi *et al.* introduced a RUSS to automatically generate the US acquisition pose for a chosen landmark point in lung and the robot is controlled to perform a wobbler movement at the acquisition point to collect diagnosis images^27^. To eliminate the need for pre-operative CT, Shida *et al.* introduced a visual servoing searching method to find the acoustic window for parasternal long-axis view of heart^28^. Recent work by Ma *et al.*^29^ introduced an anatomy-aware probe servoing framework for robotic lung ultrasound that combines visual servoing and active surface sensing to automatically navigate to standardized imaging planes while avoiding rib-induced acoustic shadows. Nonetheless, instead of acquiring one specific US frame, how to automatically generate a US scanning trajectory to fully cover and reconstruct a specific volume under ribs still requires a more intelligent and advanced algorithm. Reinforcement learning (RL), on the other hand, provides a promising alternative.2

**Fig. 2:**
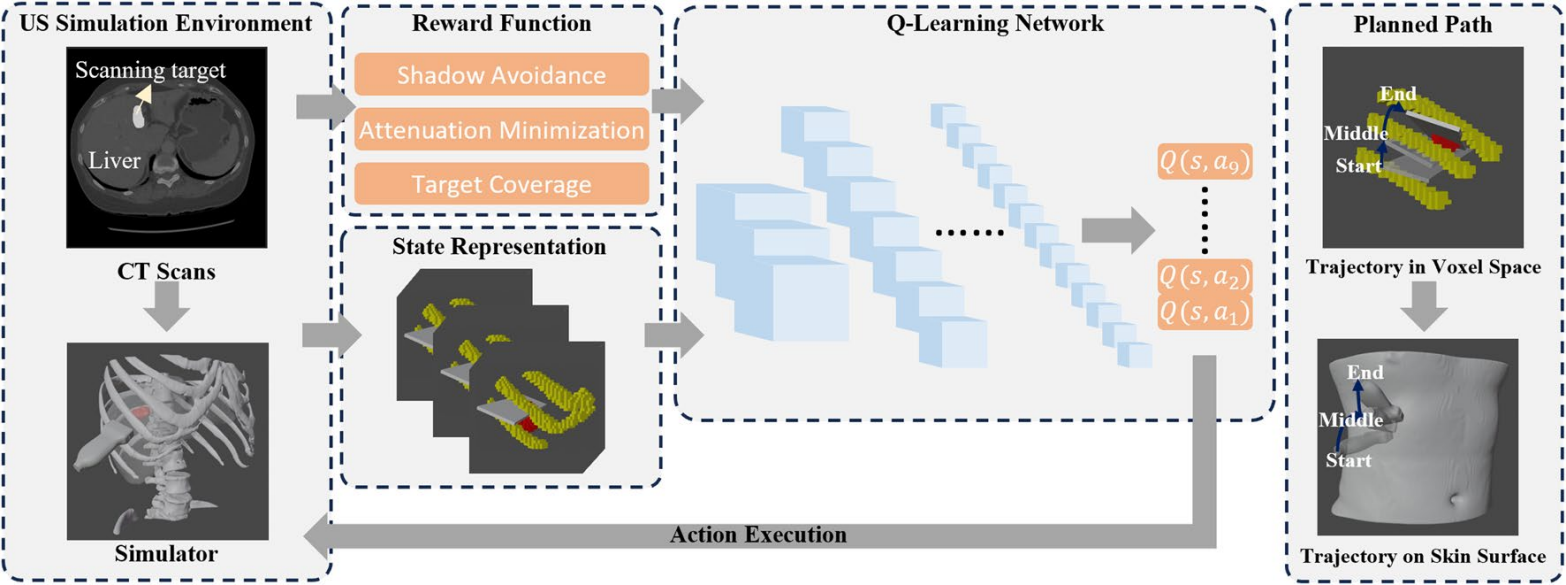
Overview of the proposed framework. The scanning target is determined by the doctors on CT and input to a simulator. The overall scanning context is depicted using a 3-channel 3D matrix. Each element within the matrix corresponds to a voxel within the space, and each channel indicates the presence of either a tumor, bone, or an ultrasound ray within the corresponding voxel. Subsequently, an RL model is employed to generate the scanning trajectory. In the end, the planned path is projected back to the skin surface of the patient.

### Reinforcement learning for RUSS

The great successes in gaming have demonstrated the superiority of RL in solving sequential decision processes^30^. Multiple efforts have been made in the field of US imaging analysis by using RL for landmark detection^31,32^, segmentation^33^, and video assessment^34^. In addition, initial attempts have also been conducted to adopt RL for RUSS. Hase *et al.* implemented an RL agent to guide a US probe for standard plane localization of spine^17^. For the similar application scenario, Li *et al.* enlarged the action space by taking the orientation into account^15^. Based on the observation from an external camera, Ning *et al.* developed an RL system to automatically locate the standard US plane^35^. To increase the generalization ability of the trained RL model, Bi *et al.* utilized the segmented masks of the anatomy of interest as inputs to bridge the gap between simulation and real environment^16^. In addition, Li *et al.* developed a learning-based navigation strategy for transesophageal echocardiography by integrating Vision Transformer with RL^36^. All the aforementioned RL approaches in RUSS primarily focus on finding one standard diagnosis plane and achieved robust performance in their specific scenarios. Nonetheless, generating a scanning path to fully cover a specific volume under ribs is yet to be fully investigated. Moreover, compared to the previous RL-based US navigation methods, in this work we try to exploit the usage of CT atlas in US scanning path planning. Such attempt has the potential to convert the partially observable Markov Decision Process to a fully observable one, thus reducing the complexity of the planning task.

## Intercostal path planning for robotic US

In order to plan a US scanning path in intercostal spaces to properly visualize internal volume of interest completely, sonographers heavily rely on prior knowledge of 3D anatomical information. Practically, we consider that it is challenging to solely use 2D US images for planning scanning path, given that US often suffers from noise, speckle, and low contrast. Therefore, in this study, we train an RL agent based on CT volumes from different patients, providing a 3D understanding of the clinical scenarios. The reasons for not using compounded US volumes are listed as follows: 1) it is hard to reconstruct a comprehensive and correct compounding volume with acoustic obstacles; 2) publicly available US volumes are very rare; 3) the image quality of US images varies a lot between different US systems. For these reasons, CT scans are utilized here to generate 3D scenery views of the rib cage and the scanning target beneath it. The trained model is then used to generate scanning trajectory for unseen patient based on patient-specific CTs. If there is no patient-specific CT available, the path planning can be done on a CT atlas and then the path is registered to the patient on-site through non-rigid registration approaches, e.g. the one specific for thoracic applications^21^.

### Environment setup

The primary target application of this study is intercostal US scanning of the right lobe of the liver. The simulation environment consists of two main components: a CT volume, serving as an anatomical representation, and a virtual US probe. Based on the provided segmentation^19^, only the skin surface, rib cage, and target liver scanning volume are retained in the simulation. The skin surface determines probe positioning, while the rib cage and scanning target are essential for evaluating the visibility of the volume of interest in US images. Notably, a complete CT segmentation is not required; instead, segmenting only the bones and skin surface suffices, which can be efficiently achieved using thresholding and filtering techniques^37^. Furthermore, only the anatomical region between the seventh thoracic vertebra and the fourth lumbar vertebra is preserved based on anatomical atlases. A virtual linear US probe is also modeled, where each probe element emits a virtual US wave. The propagation of these waves is simplified as rays that pass through soft tissues but are blocked by bones^38^, generating a visibility map of different structures. The emitted ray direction is aligned with the probe’s vertical axis. Importantly, the proposed path planning framework does not explicitly simulate US images. Instead, the US imaging plane is implicitly represented in the 3D scene visualization.

### State design

In general, the true state of the US probe navigation task is the relative pose between the US probe, the anatomy of interest, as well as the acoustic obstacles. In order to compress all these information into one state representation, CT scans of the patients are utilized. It is notable that the CT scans utilized here for training are not patient-specific. Hence, the training CTs can be any publicly available ones that hold comprehensive anatomical representatives.

**Fig. 3:**
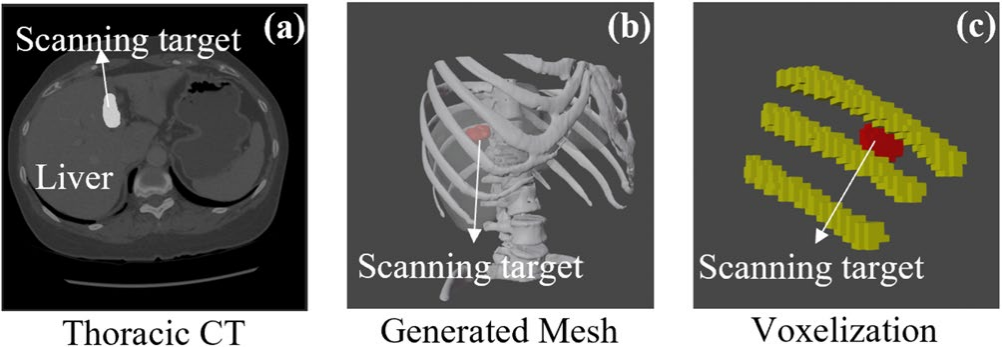
Illustrations of the voxelization process.

A representative thoracic CT is shown in Fig. 3(a). Based on the given segmentation labels, the rib cage and scanning target is transferred to mesh in 3D Slicer (https://www.slicer.org/). The shape of the scanning target here is determined by the tumors segmented from CTs or it can also be defined by the doctors manually for scanning specific volumes. Then a surrounding area of $120mm \times 120mm\times 120mm$ around the scanning target are voxelized by a resolution of 4 mm in Blender (https://www.blender.org/), where each voxel has 3 channels defined as Eq. (1)

1 $$\begin{aligned} \begin{array}{l} F[i,j,k,0] = \left\{ \begin{array}{rl} 1 & target~volume~exists~in~(i,j,k)\\ 0 & otherwise \\ \end{array}\right. \\ F[i,j,k,1] = \left\{ \begin{array}{rl} 1 & bone~exists~in~(i,j,k)\\ 0 & otherwise \\ \end{array}\right. \\ F[i,j,k,2] = \left\{ \begin{array}{rl} 1 & US~rays~pass~through~(i,j,k)\\ {0} & Bone~occlusion~occurs~or\\ & out~of~the~imaging~plane \\ \end{array}\right. \end{array} \end{aligned}$$

Here, (*i*, *j*, *k*) denote the discrete voxel indices along the *x*-, *y*-, and *z*-axes of the voxelized 3D volume. The first channel links to the volume of interest, while the second and third channels indicate the situation of bone structures and mimicked US imaging plane, respectively. Notably, as depicted in Sec. 3.1, the simulated US ray is blocked by the bones, thus generating shadows on the US imaging plane. In order to exploit the time serial information, three consecutive voxelized volumes are jointly used as the state representation in this study. Such a design of state representation is able to integrate all the necessary information in a memory-efficient way compared to using meshes directly.

### Action design

**Fig. 4:**
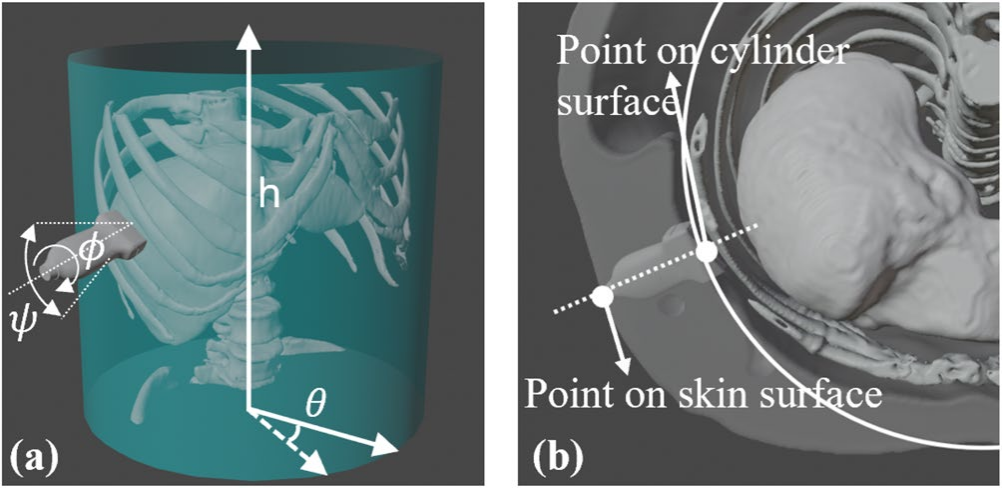
(**a**) Illustrations of the cylindrical coordinate system. (**b**) Mapping from the cylinder surface to the skin surface.

The virtual probe’s movement is constrained to the skin surface to ensure full contact. A cylindrical coordinate system is established by fitting a minimum bounding cylinder to the rib cage [Fig. 4(a)]. Since the movement is constrained on the skin surface, the virtual probe only possesses two translational degrees of freedom (DoF), which can be represented by the height (*h*) and angle ($\theta$) in the cylindrical coordinate system. To account for inter-patient rib cage variations, cylinders are affinely registered to a standard size during training and inference, with an inverse resizing process mapping the planned trajectory back to its original scale. The probe’s third translational DoF is determined by projecting it onto the skin surface along its vertical axis [Fig. 4(b)].

The proposed RL agent operates in a discrete action space with four DoFs. Two translational DoFs are controlled by moving the probe along the cylindrical surface using (*h*,$\theta$) coordinates [see Fig. 4]. The step size for *h* and $\theta$ are empirically set to 4 *mm* and $3^{\circ }$, respectively. The remaining two rotational DoFs include rotation around the probe centerline ($\phi$) and tilting along the probe’s long axis ($\psi$), both with a step size of $2^\circ$. Rotation around the short axis is excluded, as in-plane translations can achieve the same effect. To ensure feasibility in real-world scenarios, training is terminated if the probe centerline deviates more than $20^\circ$ from the skin surface normal.

**Fig. 5:**
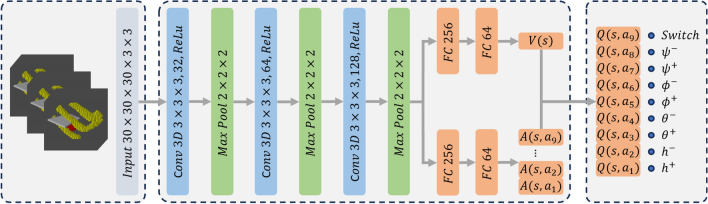
Structure of the dueling deep Q-learning network, where *h* represents the translational movements along the center line of the cylinder coordinate system, $\theta$ denotes the rotation around the cylinder center line, *phi* is the rotation angle around z-axis of the probe, and *psi* represents the rotation around the long axis of the probe footprint. $^+$ and $^-$ describe the direction of the actions.

In addition to the four DoFs, an extra ”switch” action allows the agent to toggle between readjusting and examining modes. This accounts for cases where a single intercostal gap does not fully visualize the target volume, requiring the probe to relocate to adjacent gaps for comprehensive coverage. US images obtained in readjusting mode are excluded from the 3D reconstruction to encourage the agent to search efficiently across multiple intercostal gaps before acquiring relevant imaging data. This idle mode enhances adaptability for optimal target visualization.

### Reward design

The reward is designed to encourage the agent to fully cover the target volume as fast as possible, while avoiding the intersection between the US imaging plane and the bones. To realize this, the reward function is decoupled into three aspects.

#### Coverage level of objects of interest

The agent is stimulated to cover as much target volume as possible in one sweep step. This reward part represents the most fundamental demand. The reward is then defined as Eq. (2).

2 $$\begin{aligned} r_{c} = \frac{n_t}{N} \end{aligned}$$

where *N* stands for the total number of voxels of the defined volumes of interest, and $n_t$ represents the scanned target volume in voxel at step *t*. Previously covered voxels are excluded from $n_t$, encouraging the agent to prioritize unexplored regions and accelerate convergence.

#### Attenuation minimization

Attenuation is another unavoidable characteristic of US, which is mainly related to the travelled distance of the emitted US signal^38^. To minimize the effect of attenuation on the target volume, a reward term is defined as Eq. (3)

3 $$\begin{aligned} r_{a} = e^{-\frac{d_t}{R_c}} \end{aligned}$$

where $d_t$ is the distance between the probe and the scanning target at step *t*, and $R_c$ denotes the cylinder radius (Sec. 3.3). This term encourages the agent to minimize the probe-target distance, ensuring imaging is performed from the closest intercostal area.

#### Shadow avoidance

Beyond coverage, shadow avoidance is crucial in intercostal US scanning, as bone-induced shadows hinder image interpretability. To address this, a reward function term is introduced to discourage the agent from acquiring shadow-affected US images.

4 $$\begin{aligned} r_{s} = 1-p_t = 1-\frac{n_{shadow}^t}{N_t} \end{aligned}$$

where $N_t$ refers to the total scanned volume in voxel at step *t*, and $n_{shadow}^t$ denotes the shadow volume, where simulated US rays are blocked by bones. As shown in Eq. (1), the US imaging plane is not always a complete rectangle–bone occlusions create shadows on the imaging plane. The term $p_t$ quantifies the percentage of shadowed areas within the probe’s coverage at step *t*. By negatively correlating shadow volume with the reward function (Eq. 4), the agent is penalized for acquiring shadow-affected images.

Combining all three terms, the reward is defined as follows:

5 $$\begin{aligned} r_t = r_{c}+\alpha _1 r_{a} + \alpha _2 r_{s} \end{aligned}$$

where $\alpha _1,~\alpha _2\in [0,1]$ are hyperparameters that balance the contributions of the three terms. The first term, being fundamental to task fulfillment, is always set to 1. Path planning is considered successful once $95\%$ of the target volume is scanned, at which point a high reward is assigned, as defined in Eq. (6). This coverage-based criterion reflects the clinical goal of liver tumor ablation, where surgeons aim to repeatedly scan the entire tumor region during and after ablation to monitor treatment completeness. Achieving high volumetric coverage ensures comprehensive visualization of the target area while tolerating small peripheral gaps caused by rib obstruction or acoustic shadowing. The 95% threshold was therefore chosen to represent near-complete coverage.

6 $$\begin{aligned} \begin{aligned} r_{end} = k_{end}~(1+\alpha _1 \frac{1}{D} + \alpha _2 P)\\ D = \frac{1}{T}\sum _{t=1}^{T} \frac{d_t}{R_c},~ P = \frac{1}{T}\sum _{t=1}^{T} (1-p_t) \end{aligned} \end{aligned}$$

where *P* represents the average non-shadow percentage during the scanning process, *D* refers to the average value of the normalized distance between probe and scanning target, and $k_{end}$ is a coefficient to grant the agent a high reward after a successful completion.

No rewards or penalties are accumulated in readjusting mode (Sec. 3.3). however, switching in or out of this mode incurs a one-time negative reward to discourage unnecessary transitions. To ensure shadow-free imaging, a threshold $T_{th}$ is imposed on $p_t$, the percentage of shadow volume. When $p_t$ remains below $T_{th}$ at the current step, augmented target voxels are counted, and a positive reward is given. If $p_t$ exceeds $T_{th}$, the scanned volume is disregarded, and a negative reward is assigned. This strategy trains the agent to avoid shadows during path planning. The final reward function is defined in Eq. (7).

7 $$\begin{aligned} r = \left\{ \begin{array}{cl} r_t & p_{t}<T_{th},adj=False\\ r_{end} & \sum n_t>0.95N,adj=False\\ -1 & a_{t}=switch\\ -0.1 & p_t \ge T_{th}, adj=False\\ 0 & adj=True \end{array}\right. \end{aligned}$$

$\sum n_t$ stands for the accumulated number of scanned voxels of the target, *adj* represents whether the readjusting mode is activated, and $a_t$ is the current action.

### Network design

The double dueling deep Q-learning is selected as the training pipeline. The network structure is shown in Fig. 5. The network is consisted of consecutive 3D convolutional layers with ReLU as activation function followed by max pooling layers. At the end of the convolutional process, the feature maps are flattered and fed into two streams of fully connected layers with ReLU to estimate the value function at the given state and the advantage function of all possible actions. By combining the estimations of the two branches, the Q-value function is calculated following Eq. (8) to increase the stability during the training^39^.

8 $$\begin{aligned} Q(s,a_t\mid \Theta ,\Theta ^v,\Theta ^a)=V(s\mid \Theta ,\Theta ^v)+ \left( A(s,a_t\mid \Theta , \Theta ^a)-\frac{1}{N_a}\sum _{t=1}^{N_a} A(s,a_t\mid \Theta , \Theta ^a)\right) \end{aligned}$$

where $\Theta$, $\Theta ^v$, and $\Theta ^a$ represent the trainable parameter of the main network, the state value estimation network, and the advantage value network, respectively, while $N_a$ is the dimension of the discrete action space.

## Experiments

### Implementation details

The prioritized replay is implemented to realize faster and better learning performance^40^. The learning rate is set to $7\times 10^{-5}$. The maximum number of steps in each trial is 80. The model is trained for a total number of $5\times 10^6$ steps, while the target network is updated every $5\times 10^3$ steps. The exploration rate decays from 1 to 0.05 linearly in the first $3\times 10^6$ steps. To efficiently simulate the extensive training data within a reasonable time period, we employ distributed RL across 16 training nodes. Each node is assigned with a local replay buffer with size of $5\times 10^3$.5

To train and validate the method, we manually selected 20 liver tumors and 8 rib cages from different CTs from public dataset 3D-IRCADb-01^19^. Among them, 16 tumors from 6 CT volumes were used for training, and the remaining 4 tumors from 2 CT volumes were held out exclusively for testing. The same held-out test set was consistently employed for all evaluation experiments reported in Sections 4.3 and 4.4 to ensure a fair comparison under identical anatomical conditions. In these experiments, only the initialization parameters–such as target position and number of targets–were varied, while no test samples overlapped with the training data.

To eliminate the size differences between different patients, all rib cages are affinely resized to a generic cylindrical coordinate based on the average size of human bodies in the applied dataset. Afterwards, an inverse resizing process is conducted to map the planned trajectory back to the real scale. The liver tumors in this dataset were employed as scanning targets. In each episode of training, the position, orientation and size of tumor are randomly initialized in a chosen rib cage in order to enrich the variability of training environment. The training was conducted on a PC with AMD EPYC 24-core CPU and RTX4090 GPU. The virtual environment is visualized in Blender.

### Ablation experiments

In the ablation study, the performance of different models was compared with each other by success rate, average steps, average *P*, and average *D*. If the agent can cover $95\%$ of the target volume within 80 steps, it is considered as a successful case. The average *P* and *D* are utilized to show the performance of the models on shadow avoidance and attenuation minimization, respectively. Considering the inter-patient variations, the targets of interest are further categorized into three classes based on their sizes, i.e., small (less than $4cm^3$), medium (ranging from $4cm^3$ to $13.5cm^3$), and large (exceeding $13.5cm^3$). Each ablation case (each row in Table 1) is evaluated by 100 experiments, in total 500 experiments.

**Table 1:** Results of Ablation study using Varying hyperparameter.

Hyperparameter			Success rate			Average steps			Average P ($\%$)			Average D ($\%$)		
$T_{th}$	$\alpha _1$	$\alpha _2$	S	M	L	S	M	L	S	M	L	S	M	L
1.00	1	0.5	$88\%$	$94\%$	$76\%$	14.7(7.9)	23.9(9.6)	40.0(13.7)	58.0(1.7)	60.7(2.1)	58.7(0.9)	35.6(0.4)	36.0(0.2)	36.4(0.3)
0.20	1	0.5	$95\%$	$92\%$	$81\%$	25.3(8.9)	31.4(10.2)	41.3(10.2)	92.4(0.7)	93.3(0.8)	92.2(1.3)	35.9(0.3)	36.2(0.2)	36.7(0.3)
0.05	1	0.5	$90\%$	$85\%$	$61\%$	30.5(7.1)	35.7(8.6)	47.0(12.5)	99.5(0.1)	99.1(0.2)	99.1(0.3)	36.0(0.3)	36.4(0.2)	37.3(0.3)
1.00	0	1	$90\%$	$91\%$	$76\%$	15.7(8.2)	22.4(9.4)	36.7(11.6)	61.0(1.3)	62.5(1.7)	60.7(2.0)	36.2(0.4)	36.6(0.4)	36.9(0.4)
1.00	2	0	$94\%$	$87\%$	$80\%$	14.3(7.5)	22.9(9.3)	37.1(12.4)	58.7(1.9)	58.5(2.3)	60.1(1.7)	35.3(0.3)	35.7(0.3)	35.9(0.4)

mean(std)

#### Threshold parameter of shadow region

Firstly, the ablation study was conducted to validate the effect of threshold value $T_{th}$ in reward function Eq. (7). If the portion of shadow volume $p_t$ at current step is larger than the threshold value $T_{th}$, a negative reward will be assigned to the agent to increase the shadow awareness of the trained agent. The other hyperparameters were set as the default values, i.e., $\alpha _1=1$, and $\alpha _2=0.5$. Here, $T_{th}$ represents the maximum tolerated proportion of shadowed area in the ultrasound imaging plane. Three representative settings were evaluated: $T_{th}\in \{{1.00}, {0.20}, {0.05}\}$, corresponding to no constraint, moderate constraint (20 % shadow allowed), and strict constraint (5 % shadow allowed), respectively.

As shown in the first three rows of Table 1, the model trained with $T_{th}=0.20$ surpasses the performance of the other two models in most cases across all tumor sizes in terms of success rate. The only exception happens when $T_{th}=1.00$ for the medium-size tumor ($92\%$ vs $94\%$). Interestingly, these results are somehow counter-intuitive. One would have expected that the agent trained with $T_{th}=1.00$ to yield the highest success rate since it possesses no constraint at all. Such intuition holds true during the training phase, the model with $T_{th}=1.00$ has the highest success rate of $95\%$ on training sets. However, it fails to generalize on unseen tumor shapes and rib cages during the test phase, resulting in a diminished success rate compared to its training performance. If the agent is not sufficiently constrained regarding the scan quality during training, it cannot learn the occlusion relationship between the ribs and tumors efficiently.

When the threshold value increases, the average *P* of each model also rises since the essence of $T_{th}$ is to impose a hard constraint to prevent the existence of shadows during trajectory planning. Apart from the average percentage of non-shadow volume *P*, the average steps and average distance *D* between probe and target on cylindrical coordinate also increase under a higher threshold value. With the increasingly severe constraint, it is reasonable for the agent to perform extra steps to find an appropriate acoustic window and visualize the target volume from a distance to avoid intersection with bones.

#### Hyperparameters of attenuation minimization and shadow avoidance

The reward function consists of three terms: the target coverage term, the attenuation minimization term, and the shadow avoidance term. As shown in the last two rows of Table 1, when the attenuation minimization term ($\alpha _1$) is removed from the reward function, the average *D* increases in all three different target sizes compared with standard hyperparameter setup ($T_{th}=1.00$, $\alpha _1=1$, $\alpha _2=0.5$). The increase in average *D* is even more significant when comparing it with setting $\alpha _1$ to two and setting $\alpha _2$ to zero (fifth row of Table 1), which demonstrates the effectiveness of explicitly considering the attenuation minimization term ($\alpha _1$) in the reward function. For the case when the shadow avoidance term is restrained, the average portion of non-shadow volume decreases compared to the case when only shadow avoidance term is included. Thus, it can be concluded that the attenuation minimization term and the shadow avoidance term are able to achieve the expected effects, respectively.

**Fig. 6:**
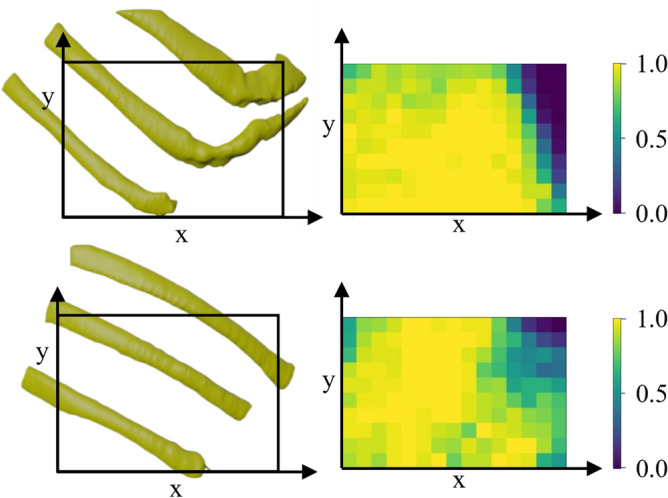
Heatmaps of success rates based on scanning target positions in the X-Y plane defined in CT coordinate.

### Impact of target locations

To investigate the impact of the target positions on success rate, ten segmented tumors are initialized at the center point of every voxel beneath the ribs, where the target tumor has no intersection with the ribs. The success rate is computed for each possible configuration. The model trained with the hyperparameter configuration of $T_{th}=0.20$, $\alpha _1=1$, and $\alpha _2=0.5$ was employed on two unseen ribs in this study.

Since the position of the target tumor along the z-axis in CT coordinate (spanning from front to back) does not have a big impact on the success rate compared to x- and y-axis, therefore, the success rates over the z-axis were averaged and displayed on the x-y plane as shown in Fig. 6. It can be seen that regions with narrower intercostal spaces exhibit a relatively lower success rate compared to other areas. For instance, the success rate in the upper right section of the first ribcage is notably low, where the intercostal space between ribs is only 10*mm*. This compact spacing poses a significant challenge for the agent to scan these areas effectively while still achieving a relatively high proportion of non-shadowed areas ($T_{th}=0.20$) 2.

**Table 2:** Performance of the proposed RL model on multiple targets scanning task.

Number of targets	Success rate		Average steps		Average P ($\%$)		Average D ($\%$)	
	S	M	S	M	S	M	S	M
1	$95\%$	$92\%$	25.3(6.1)	31.9(7.5)	95.6(0.4)	95.2(0.4)	36.6(0.4)	36.8(0.4)
2	$91\%$	$86\%$	37.3(10.9)	44.7(9.6)	95.2(0.8)	94.8(0.9)	37.3(0.2)	37.5(0.2)
3	$90\%$	$84\%$	42.9(9.7)	51.4(10.1)	95.0(0.7)	94.6(0.9)	37.5(0.3)	37.7(0.3)

mean(std)

### Results on multiple scanning targets

Considering that lesions could be located in various areas, especially if the tumor was incompletely removed after resection. It would also be important to ascertain if the proposed framework can effectively operate in configurations with multiple scanning targets. Therefore, we randomized the number of targets from 1 to 3 and retrained a policy for scanning multiple targets. To reduce the probability of targets overlapping, the large-sized target was not used in this validation. During the training, the number of targets was randomly selected at initialization. The hyperparameter configuration adopted here was $T_{th}=0.20$, $\alpha _1=1$, and $\alpha _2=0.5$ as well. The results are shown in Table 2. The success rate drops as the number of targets increases. However, the trained model can still deliver decent success rates for both small- and medium-sized targets with $90\%$ and $84\%$ respectively. A slight drop in success rate is observed when the number of scanning targets increases. This behaviour is expected, as multi-target planning introduces additional spatial and acoustic constraints that require the agent to generate longer and more complex trajectories. The increased inter-target distance and cumulative risk of rib-induced shadowing can lead to occasional partial coverage. Nevertheless, the moderate decrease in success rate demonstrates that the learned policy remains robust and capable of handling complex multi-target scenarios.

## Conclusion and discussions

This work presents an RL-based approach for automatically generating US scanning paths to fully cover and reconstruct single or multiple target volumes in intercostal spaces while minimizing acoustic attenuation and shadowing. Training is conducted in a simulation environment using a CT atlas. Unlike US image-based navigation, directly using anatomical models as state representation ensures full observability during navigation. The ablation study has validated the effectiveness of the proposed framework (success rate: $95\%$, $92\%$, and $81\%$ for unseen small-, medium-, and large-sized target; success rate: $90\%$ and $84\%$ for three small- and medium-sized targets).

This work marks the initial step in developing a fully autonomous US scanning system for intercostal applications. A comprehensive autonomous US scanning system for liver should consist of different components, e.g., path planning module, registration module, correction module and robot control module. The planned scanning path is registered to the patient on-site and the robot is employed to execute the scanning while ensuring appropriate contact and force interaction with the surface. By applying non-rigid registration approaches^20,21^, we can then plan the trajectory on a CT atlas and realize non-patient-specific path projection. A correction module will then refine the trajectory to compensate for residual registration errors, anatomical deviations, or patient repositioning by performing local adjustments^29,41^ or real-time re-registration^25^ based on intra-operative perception. One possible application scenario for such robotic system is liver ablations. The robot can then help the surgeons to monitor the target tumor as a whole during and after the ablation to assess the surgical outcomes.

This work focuses on the development of a robust path planning module as a fundamental component within a broader autonomous ultrasound framework. The current validation is conducted in a CT-based simulation environment to quantitatively assess trajectory feasibility, completeness, and shadow avoidance under anatomically realistic conditions. Although soft-tissue deformation, respiratory motion, and probe–tissue interaction are not yet modeled, the simulation provides a controlled and reproducible setup for evaluating planning efficiency. Future work will integrate the proposed policy with real-time motion compensation^42,43^ and force-controlled robotic execution^44^ to account for breathing-induced movements and ensure stable acoustic coupling during validation on real robotic platforms and patient data. In addition, the present study employs a simplified ultrasound simulation that models acoustic shadows and attenuation geometrically. While this enables efficient training and evaluation, it does not reproduce full ultrasound image formation, including noise and scattering. Future work will incorporate more realistic ultrasound simulation to assess the robustness of the framework under image-level variability.

## Data Availability

The source code is available at: https://github.com/yuan-12138/Intercostal-US-RL. Data will be made available on request.
